# Effect of Stabilizers on Encapsulation Efficiency and Release Behavior of Exenatide-Loaded PLGA Microsphere Prepared by the W/O/W Solvent Evaporation Method

**DOI:** 10.3390/pharmaceutics11120627

**Published:** 2019-11-24

**Authors:** Heejun Park, Dong-Hyun Ha, Eun-Sol Ha, Jeong-Soo Kim, Min-Soo Kim, Sung-Joo Hwang

**Affiliations:** 1 College of Pharmacy, Pusan National University, 63 Busandaehak-ro, Geumjeong-gu, Busan 46241, Korea; pharmacy4336@pusan.ac.kr (H.P.); biz_magic@naver.com (D.-H.H.); edel@pusan.ac.kr (E.-S.H.); 2 Dong-A ST Co., Ltd., Giheung-gu, Yongin, Gyeonggi 446-905, Korea; ttung2nd@naver.com; 3 College of Pharmacy and Yonsei Institute of Pharmaceutical Sciences, Yonsei University, 85 Songdogwahak-ro, Yeonsu-gu, Incheon 21983, Korea

**Keywords:** exenatide, stability, PLGA microsphere, encapsulation efficiency, in vitro release

## Abstract

The aim of this study was to investigate the effects of various stabilizers on the encapsulation efficiency and release of exenatide-loaded PLGA (poly(lactic-*co*-glycolic acid)) microspheres prepared by the water-in-oil-in-water (W/O/W) solvent evaporation (SE) method. It was shown that the stabilizers affected exenatide stability in aqueous solutions, at water/dichloromethane interfaces, on PLGA surfaces, or during freeze-thawing and freeze-drying procedures. Sucrose predominantly reduces instability generated during freeze-thawing and freeze-drying. Phenylalanine prevents the destabilization at the water–dichloromethane (DCM) interface through decreased adsorption. Poloxamer 188 enhances stability in aqueous solutions and prevents adsorption to PLGA. Proline and lysine decrease adsorption on PLGA surfaces. Fourier transform infra-red spectroscopy (FT-IR) was used to find the molecular interaction of additives with exenatide or PLGA. Additives used in stability assessments were then added stepwise into the inner or outer water phase of the W/O/W double emulsion, and exenatide-loaded microspheres were prepared using the solvent evaporation method. The effect of each stabilizer on the encapsulation efficiency and release behavior of microspheres correlated well with the stability assessment results, except for the negative effect of poloxamer 188. Particle size analysis using laser diffractometry, scanning electron microscopy (SEM), water vapor sorption analysis, differential scanning calorimetry (DSC), and circular dichroism (CD) spectroscopy were also employed to characterize the prepared exenatide-loaded PLGA microsphere. This study demonstrated that an adequate formulation can be obtained by the study about the effect of stabilizers on peptide stability at the preformulation step. In addition, it can help to overcome various problems that can cause the destabilization of a peptide during the microsphere-manufacturing process and sustained drug release.

## 1. Introduction

Many peptide drugs have been developed into commercialized medicines using PLGA (poly(lactic-*co*-glycolic acid)) microsphere technology to enable long-term application through sustained release. For such development, stability improvements to avoid peptide degradation and to ensure optimized sustained drug release are of particular interest [1]. Peptide instability can be induced by exposure to various environmental stresses, such as high temperature, pH, light, adsorption, salts, metal ions, shear stress, freezing, drying, and organic solvent. Many strategies have been suggested to improve peptide stability, including the addition of a stabilizer, encapsulation, structural modification, or complexation with biocompatible molecules, and various micro-/nanoparticulate drug delivery systems [2]. The basic characteristics of a peptide should be completely understood to develop a final formulation for a successful peptide-injectable dosage form. In particular, a stability assessment on the effects of additives should be performed at the stage of preformulation [3]. The stabilities of encapsulated peptides are not equivalent to those of other biomaterials, even when the same amount of either drug is used. In addition, in order to solve the urgent instability problem of current marketed peptide medicines, the best way may be to improve by using FDA (Food and Drug Administration)-approved and easily applicable inactive ingredients. If information on the stabilizing effect of the additives is obtained at the preformulation step in advance, it can be applied quickly to settle an instability issue. 

Microencapsulation of peptides, such as PLGA microspheres, is generally designed to both counteract the instability of peptides and achieve sustained release over a prolonged period time. Multiple types of microsphere-manufacturing techniques have been developed, including the widely used extraction or evaporation of water-in-oil-in-water double emulsion (W/O/W) technique [4]. In addition, the drying process for producing the final formulation in a solid state has a very large impact on stability. The additives and compositions of each phase in double emulsion are critical factors for determining drug stability during the manufacturing process, drug release, and storage [5]. For example, an initial burst release followed by an incomplete release is frequently observed due to drug instability. They are common topics for research into sustained release formulations. The burst release problems could result from an undesirable peptide instability, like process-induced adsorption of the drug onto the surface of microspheres, due to poorly designed formulations. Moisture-induced aggregation and ionic interactions also often occur in the initial phase of the microsphere hydration. Later, incomplete release following initial burst release is frequently observed due to instability by acylation of peptides and PLGAs, with peptide adsorption to PLGA believed to be a common precursor to peptide acylation. During polymer erosion, non-specific protein adsorption onto surface areas that trigger degradation and covalent/noncovalent aggregation due to the formation of acidic PLGA degradation products is responsible for incomplete drug release [6]. As a result of difficulties in peptide stabilization in the PLGA microsphere, many strategies have been examined to improve peptide stabilization, but no prefect strategy currently exists to avoid instability issues [7]. 

The manipulation and optimization of various formulation factors is required to successfully develop new formulations of peptide drugs with desirable pharmaceutical properties. Such refinement should be carried out at very early stages of development using PLGA microsphere formulations to ensure sustained delivery of new peptide drugs as stated above. The properties of PLGA microspheres for different drugs may be highly variable, especially for mid-length peptides, like exenatide. To obtain an optimized formulation for the preparation of peptide-loaded PLGA microspheres, multiple factors affecting the formulation should be considered, including the pH of the inner/outer aqueous phases and the type and amount of stabilizers [8]. Furthermore, peptide adsorption to PLGA is a critical factor affecting peptide release kinetics from PLGA microspheres. Indeed, variations in surface activity and properties have been widely used to inhibit peptide adsorption to hydrophobic polymers [9]. Finally, formulations must undergo extensive testing to identify the optimal parameters and release characteristics and evaluate effectiveness.

Exenatide instability and related problems in its commercial manufacture and clinical usage have been widely reported [10]. For example, adverse effects like vomiting may increase with higher rates of exenatide burst release [11]. Many groups are currently attempting to suppress exenatide burst release and the incomplete release of commercial products [12,13,14,15,16,17,18,19,20,21,22,23,24,25,26,27,28,29,30,31]. Although many investigations into exenatide formulations and dosages have been performed, there is still a lack of useful information about exenatide stability. 

The aim of this study was to show how useful the evaluation of stabilizer effects in the preformulation stage is for the optimized design of peptide-loaded PLGA microsphere formulation and improvement of the final product quality. Exenatide was used as a model peptide (Figure S1a). We examined the effects of a variety of components, such as carbohydrates, amino acids, and amphipathic surfactants, as stabilizers on the stability of exenatide in aqueous solution and the water-in-oil (W/O) interface. The effects of additives on the adsorption of exenatide to PLGA were also examined. The stabilizers used in the experiments are all excipients currently available for injections under FDA approval. The stabilizers were selected based on the related literatures and the results of stability evaluations, and PLGA microspheres were prepared with the addition of selected stabilizers to each phase of double emulsion according to the purpose and their characteristics [32,33,34,35,36,37,38]. Finally, after evaluation of the encapsulation efficiency (EE) and release behavior of exenatide-loaded PLGA microspheres (ELPM) prepared by the W/O/W solvent evaporation (SE) method, we also looked for a rational correlation of the stability test results as pre-formulation procedures with the stability of exenatide during microsphere preparation and drug release.

## 2. Materials and Methods 

### 2.1. Materials

Exenatide was purchased from American Peptide Company, Inc. (Sunnyvale, CA, USA). PLGA 50/50 was obtained from Boehringer-Ingelheim (Resomer 503H, Ingelheim am Rhein, Germany). Sucrose, lysine, phenylalanine, proline, and poly(vinyl alcohol) (PVA, M.W. 13,000–23,000) were purchased from Sigma Aldrich (Saint Louis, MO, USA). Poloxamer 188 was kindly supplied by BASF (Lutrol^®^F68, Ludwigshafen, Germany) and 1,2-dimyristoyl-sn-glycero-3-phosphocholine (DMPC) was obtained from Corden Pharma (Liestal, Switzerland). Acetonitrile, trifluoroacetic acid (TFA), and dichloromethane (DCM) were purchased from Merck (Fair Lawn, NJ, USA). All other chemicals were of reagent grade and used without further purification.

### 2.2. Quantification of Exenatide

Reverse phase high performance liquid chromatography (RP–HPLC) was used to analyze exenatide content. HPLC analysis was performed using an Agilent 1290 Infinity HPLC system (Waldbronn, Germany) consisting of a pump (Model 1260 Quat Pump VL), an auto sampler (Model 1260 ALS), and UV detector (Model 1260 VWD DL) as previously described with slight modifications [14]. The autosampler and column temperature were maintained at 5 and 30 °C, respectively, during analysis. The mobile phase was composed of 40% acetonitrile (ACN) with 0.1% TFA/60% water with 0.1% TFA. RP-HPLC was conducted using a C18 column (Kintex C18 2.6 µm, 100Å, 4.6 × 100 mm, Phenomenex Inc., Torrance, CA, USA). UV detection was performed at 210 nm. Columns were eluted at a flow rate of 1.5 mL/min. The injection volume was 10 µL. 

### 2.3. Stability Tests

The conditions of the stability evaluations were set in consideration of the physicochemical properties of exenatide (Figure S1a) and the actual environment used in the microsphere preparation and the in vitro release test. A number of candidate additives were screened through a preliminary study. All of the used additives are clinically used in injectable dosage forms under current FDA approval. Only those with significant effects were included in this paper. An evaluation of exenatide stability in aqueous solutions with or without additives was performed. Test solutions of exenatide were prepared to a concentration of 0.1 mg/mL in pH 4.5 acetate buffer. The final concentration of hydrophilic additives, such as sucrose and poloxamer 188, was 40 µg/mL. The final concentration of amino acid additives (proline, lysine, and phenylalanine) was 0.1 mM. After stability tests, samples were centrifuged at 12,000 rpm and 5 °C for 10 min. The concentration of exenatide in the collected supernatant was determined by RP-HPLC. Exenatide recovery percentages after stability tests were calculated by dividing the initial concentration by the post-test concentration and then multiplying by 100. 

#### 2.3.1. Aqueous Solution Stability of Exenatide

The test solutions of exenatide (0.1 mg/mL) containing hydrophilic additives were prepared in pH 4.5 acetate buffer. They were transferred to a certified low adsorption amber glass vial (Supelco, Sigma Aldrich, Bellefonte, PA, USA), and then sealed with a cap. A pH condition of 4.5 was selected because this pH was used favorably for the marketed injectable liquid formulation of exenatide considering the stability and compatibility with PLGA [16,39,40]. These vials were then placed into a water bath maintained at a constant temperature of 50 °C for 36 h (Supplementary Materials, Figure S2a).

#### 2.3.2. W/O Interface-Induced Stability

Emulsification of an aqueous exenatide solution with DCM was performed to simulate the W/O interface-induced stress during the formation of W/O emulsion with a slight modification of previously reported methods [33,41,42]. To test the effects of hydrophilic additives, 1 mL of test solutions (0.1 mg/mL of exenatide, pH 4.5 acetate buffer) containing hydrophilic additives was gently poured into 10 mL of dichloromethane (DCM). Alternatively, the selected amphipathic additives (nonionic surfactant; poloxamer 188 and phospholipid; 1,2-dimyristoyl-sn-glycero-3-phosphocholine (DMPC)) were dissolved in DCM to a final concentration of 2.5 mg/mL and then 1 mL of test solution without additives was gently added to 10 mL of additive-containing DCM solution. After that, the mixture was homogenized by using a homogenizer (T-18 Basic ULTRA-TURRAX^®^, IKA^®^-WERKE GMBH & Co. KG, Staufen, Germany) at 21,500 rpm for 15 min. After homogenization, 4 mL of pH 4.5 acetate buffer was added then samples were centrifuged at 3000× *g* for 20 min to complete phase separation. After collecting as much water phase as possible, residual DCM was further removed by using a rotary vacuum evaporator (N-1110, EYELA, Bohemia, NY, USA) at ambient condition for 1 h (Supplementary Materials, Figure S2b). 

#### 2.3.3. Freeze-Thawing Stability

A test solution volume of 1 mL (0.1 mg/mL of exenatide, pH 4.5 acetate buffer) was transferred to a CryoTube^TM^ (Nunc, Thermo fisher Scientific, Loughborough, UK) then frozen at −70 °C for 12 h. The frozen samples were then thawed at 10 °C for 1 h. This freeze-thawing process was repeated 7 times for each sample (Supplementary Materials, Figure S2c).

#### 2.3.4. Freeze-Drying Stability

The method for the preparation of the frozen samples was the same as that used for the above freeze-thawing stability experiments. Frozen samples in CryoTubes^TM^ (Nunc, Thermo fisher Scientific, UK) were lyophilized using a freeze dryer (Model FD8508, IlshinBioBase Co., Ltd., Gyeonggi-do, Korea) at 50 mTorr of vacuum for 3 days. Lyophilized samples were then dissolved in 1 mL of DW (Supplementary Materials, Figure S2d).

### 2.4. Adsorption Tests of Exenatide to PLGA

The adsorption of exenatide was evaluated as previously described in the literature with a slight modification [34]. The effects of additives on the adsorption of exenatide to the PLGA polymer were investigated by quantifying the amount of peptide adsorbed onto a PLGA polymer layer. To obtain the PLGA polymer layer, 700 µL of PLGA 50/50 solution (Resomer 503H, Boehringer-Ingelheim, Germany) at a concentration of 80 mg/mL in DCM were transferred to individual wells of a polystyrene 24-well plate (Thermo Scientific, Waltham, MA, USA). Immediately, 400 µL of PLGA solution were removed. The visual observation confirmed the side walls of the wells to be coated. After evaporation under a hood for 24 h, the plate was placed under vacuum to dry for 48 h, and then the polymer film annealed at a temperature of 55 °C for 3 h to create a smooth surface. 

#### 2.4.1. Effect of pH in the Aqueous Phase

Test solutions (0.1 mg/mL of exenatide) without additive were prepared using various buffered solutions with pH conditions of 3.5 (30 mM citrate buffer), 4.5 (30 mM acetate buffer), and 7.4 (phosphate-buffered saline, PBS). A 100-µL volume of prepared test solution was added to each PLGA-coated well, and then the 24-well plate was sealed with sealing film. After shaking at 37 °C for 12 h, the amount of free exenatide was quantified using RP-HPLC (Supplementary Materials, Figure S2e). The percentage of adsorbed exenatide was calculated by subtracting the post-test concentration from the initial concentration and dividing by the initial concentration and finally multiplying by 100.

#### 2.4.2. The Effect of Hydrophilic Additives in the Aqueous Phase

To study the effect of hydrophilic additives on exenatide adsorption to PLGA film, 100 µL of test solution (acetate buffer, pH 4.5) containing hydrophilic additives were added to a PLGA-coated well. The concentration of hydrophilic additives was same as above stability test. For the amino acids, tests were also conducted in DW and pH 7.4 PBS. The pH 4.5 acetate buffer and water were chosen as aqueous phases taking into account the environment of the internal aqueous phase and outer aqueous phase during microsphere preparation, respectively. A pH of 7.4 was selected considering the pH condition of the in vitro release tests. Subsequent procedures were performed in the same manner as the above adsorption experiments with varying pH. 

#### 2.4.3. The Effect of Amphipathic Additives Blended into PLGA

The selected amphipathic additives (30 mg/mL, Poloxamer 188 and DMPC) were dissolved in DCM solution with a PLGA concentration of 80 mg/mL. PLGA films containing hydrophobic additives were prepared using the same method as with blank PLGA film. A total of 100 µL of test solution (pH 4.2) without additive were transferred to a PLGA film. Subsequent procedures were performed in the same manner as the above experiments with hydrophilic additives (Supplementary Materials, Figure S2e).

### 2.5. Preparation of Exenatide-Loaded Microspheres

Exenatide-loaded microspheres were prepared by the double emulsion (W/O/W)–solvent evaporation method. Various formulations of exenatide-loaded microspheres are presented in Table 1. Briefly, 10 mg of exenatide powder was dissolved in 0.1 mL of 30 mM acetate buffer (pH 4.5) with or without additives. Aqueous exenatide solution was mixed with 2.5 mL DCM containing 185 mg of PLGA polymer and emulsified using a homogenizer (T-18 Basic ULTRA-TURRAX^®^, IKA^®^-WERKE GMBH & Co. KG, Germany) at 21,500 rpm for 5 min in an ice bath. This primary emulsion was then added to 12 mL of an outer aqueous phase containing 1% PVA (poly(vinylalcohol)) with or without 0.1 M lysine. The emulsification continued at 6500 rpm for 1 min. An additional 12.5 mL of outer aqueous phase was poured slowly into the secondary (W/O/W) emulsion. The DCM in the double emulsion was removed using a rotary vacuum evaporator (N-1110, EYELA, USA) at 35 °C. The resulting microspheres were washed three times with a 5% dextrose and 0.9% NaCl solution by centrifugation at 600× *g* (Eppendorf centrifuge 5019R, Hamburg, Germany), and then freeze-dried by the same lyophilization method as used above for the freeze-drying stability study. 

### 2.6. Characterization of Lyophilized Microsphere Morphology

Scanning electron microscopy (SEM, JSM-6700F, JEOL, Hiroshima, Japan) was used to observe the morphology of the PLGA microsphere after freeze drying. Microspheres were placed on a metal sample stub using conductive carbon double-sided adhesive tape. Gold-palladium coating was applied using an automatic sputter coater. The morphology and appearance of samples were examined using a SEM at an accelerating voltage of 5 kV.

### 2.7. Particle Size Analysis

The distribution of particle sizes was determined using laser diffractometry with a Mastersizer 2000 containing a Hydro 2000S module (Malvern Instrument, Malvern, UK) for wet sample dispersion. Particle sizes are expressed as the volume mean diameter (VMD) in micrometers (µm). The size distribution was determined using the apan value, which is calculated as the ratio of D90%–D10% to D50%, where DN% indicates the volume particle diameter at each cumulative volume percentage. Smaller span values represent narrow particle size distributions.

### 2.8. Drug Loading Capacity (LC) and Encapsulation Efficiency (EE)

A total of 10 mg of lyophilized microspheres were dissolved in 1 mL of acetonitrile, to which 1 mL of 30 mM acetate buffer solution (pH 4.5) were added. The mixture was centrifuged (1730 MR, GYROZEN Co., Ltd., Daejeon, Korea) at 12,000 rpm for 5 min. The supernatant was then collected and injected into an RP-HPLC system and the amount of exenatide in dried microsphere was determined. The loading capacity (LC) and encapsulation efficiency (EC) for the direct analysis method were calculated using the following equations:

LC (%) = (Weight of exenatide in microsphere/Weight of microsphere) × 100,(1)

EC (%) = (Measured LC/Theoretical LC) × 100.(2)

### 2.9. In Vitro Release of Exenatide

The phosphate buffer saline (PBS, pH 7.4) containing 0.02% (*w/v*) Tween 20 and 0.01% sodium azide was used for the in vitro release test. A total of 20 mg of microspheres were weighed then added into 2 mL of dissolution medium and incubated in a shaking water bath at 37 °C under continuous agitation (100 rpm) for 24 days. At predetermined time intervals, samples were centrifuged at 4000 rpm for 5 min. An aliquot of the resulting supernatant was then collected, and an equal volume of fresh release medium was added to the release tube. The supernatant was then centrifuged at 12,000 rpm (5 °C) for 10 min and the amount of exenatide in the supernatant was determined by RP-HPLC. The percentage of exenatide measured on the first day was considered the initial burst release (IBR) and used for comparison between samples.

### 2.10. Water Vapor Sorption Analysis

Water vapor sorption analysis was also conducted in a humidity-controlled microbalance (DVS, Surface Measurement Systems, Wembley, UK) to evaluate the moisture sorption/desorption behavior. Each sample was placed on the sample pan, which was equilibrated to 0% relative humidity (RH). The experimental cycle consisted of a sorption cycle followed by a desorption cycle. The RH was increased from 0% to 95% RH in steps of 5% RH for the adsorption isotherm and decreased from 95% to 0% RH in steps of 5% for the desorption isotherm. At each relative humidity step, the system controlled the RH and monitored the sample weight until it reached equilibrium conditions. The equilibrium weight and temperature at the RH step were then recorded.

### 2.11. Differential Scanning Calorimetry (DSC)

DSC measurements were carried out using a DSC S-650 (Scinco, Ltd., Seoul, Korea). Calibration was performed first with indium (melting point = 156.6 °C) and zinc (melting point = 419.5 °C) standards at a heating speed of 10 °C/min. Accurately weighed 2-mg samples were placed in aluminum pans, and then sealed with an aluminum lid. A sealed empty pan was used as a reference. Measurements were conducted using a heating rate of 10 °C/min under a nitrogen purge with a flow rate of 20 mL/min.

### 2.12. Fourier-Transform Infrared (FT-IR) Spectroscopy

FT-IR analysis was conducted using a Nicolet 380 FT-IR spectrometer (Thermo Fisher, Waltham, MA, USA) equipped with an attenuated total reflectance (ATR) accessory. Spectra were collected from 400 to 4000 cm^−1^ at a 4 cm^−1^ resolution and 256 scan numbers. Prior to the collection of spectra for the samples, background spectra were scanned first by purging the detector with nitrogen gas to minimize interference by water vapor and CO_2_. Second derivative data were obtained by OriginPro 8 software, (OriginLab Corp, Northampton, MA, USA) and then processed with 7-point smoothing.

### 2.13. Circular Dichroism (CD)

CD spectroscopy (Jasco J-810 spectrometer, Jasco, Tokyo, Japan) was exerted to investigate the secondary structure of exenatide in PLGA microsphere. The exenatide was extracted from microspheres by dissolving about 10 mg of exenatide microspheres in 3 mL acetonitrile followed by the addition of 3 mL of distilled water. The presence of acetonitrile from the solution was removed by rotary evaporation under vacuum. After that, samples were centrifuged (1730 MR, GYROZEN Co., Ltd., Korea) at 12,000 rpm for 5 min. Supernatant was collected as sample solution. Unprocessed raw exenatide was used for a comparison of the secondary structural integrity. The detection was performed using a 0.1-cm path cuvette with a scan rate of 50 nm/min at 30 °C, with 24 scans averaged to generate each spectrum. For comparison, unprocessed exenatide dissolved in distilled water was analyzed by CD spectroscopy in the same way. 

## 3. Results and Discussion

### 3.1. Effects of Additives on Various Stability of Exenatide

#### 3.1.1. Aqueous Solution Stability

Peptides can be degraded through hydrolysis and temperature-dependent destabilization. The recovery of exenatide for samples without additives was only 34.2%. The test temperature of 50 °C probably induced acceleration of exenatide destabilization in aqueous solution. Significant improvements in aqueous exenatide stability were observed from all additives (Table 2). Many research papers have reported the effect of excipients on the peptide stability in aqueous solution to understand the stabilization mechanism [43]. Some hydrophilic additives are depleted in the surface layer of peptide in solution because they increase the surface tension of water by the formation of cooperative structures with water molecules. This preferential surface exclusion of the additive results in preferential hydration of peptides and increases the free energy of the system consisting of peptide, additive, and water. Peptides in solutions can be stabilized by the presence of additive because peptide denaturation increases this thermodynamically unfavorable effect and peptides in a solution state prefer to maintain their native conformation [3]. This theory, called preferential interaction, is the widely accepted mechanism of peptide stabilization in aqueous solution. In particular, sugars, such as sucrose (Figure S1c), are known to stabilize peptides in an aqueous solution, and their stabilizing effect is mainly controlled by this preferential interaction mechanism [7,8]. 

Among the water-soluble additives used in this study, poloxamer 188 had the largest effect. This may be due to synergic effects by other complex mechanisms as well as preferential interaction. Peptides can be adsorbed to air/water interfaces followed by subsequent reorientation and rearrangement of the adsorbed molecule. Nonionic surfactant, like poloxamer 188, can decrease the surface tension and induce a decrease in peptide adsorption at the air/water interfaces, thereby reducing changes in the secondary structure and destabilization of the peptide [3]. In addition, aggregation at the hydrophobic surface of peptides can be prevented by their micelle formation property. The formation of micelles can also decrease peptide hydrolysis as the chance of exposure to acidic or basic molecules is inhibited [44]. 

The stabilization effect of amino acids, including proline (Figure S1d), lysine (Figure S1e), and phenylalanine (Figure S1f), was shown in this study [38]. These amino acids were relatively less effective than poloxamer 188 (Figure S1g) and sucrose, but significantly improved the aqueous stability of exenatide. Amino acids, including proline, lysine, and phenylalanine, can stabilize peptides most likely through preferential interaction. In addition, proline can form amphipathic supramolecular assemblies that prevent protein aggregation. The hydrophobic, aromatic amino acid phenylalanine tends to reside in hydrophobic interiors or the transmembrane segments of peptides. In such positions, it can play a diverse role in peptide stabilization.

#### 3.1.2. W/O Interface-Induced Instability

Exenatide recovery after the W/O interface stability tests was 59.4%, confirming that exenatide instability can be caused by W/O emulsification during PLGA microsphere preparation. The hydrophobic surface of DCM makes the circumjacent water molecule become highly ordered. The surrounding water molecules that stabilize the peptide can be dehydrated at the W/O interface, thus the peptide molecule can contact the DCM directly. This peptide adsorption to the W/O interface spontaneously displaces ordered water and induces chemical and physical destabilization of peptides because this dehydration increases entropy [45]. Except for sucrose, all aqueous phase-soluble additives reduced W/O interface-induced instability. Stabilization effects at the DCM/water interface are related to the affinity of the additive for the methylene chloride/water interface. If the additive molecule has an affinity for the DCM/water interface through its surface activity, it is able to replace the role of water, hence preventing the adsorption of peptide molecules to the DCM/water interface [33]. 

In particular, phenylalanine, with a hydrophobic phenyl group, showed dramatic stabilizing effects, which may be due to the replacement of water molecules by its good affinity for the DCM/water interface. 

Lysine and proline showed a small effect. This may be due to their low surface activity considering their chemical structure without a hydrophobic moiety. The W/O interface-induced instability was not significantly reduced by the addition of sucrose (*p* > 0.05). This may be due to the lack of its surface activity and affinity for the DCM/water interface. Several studies also presented similar results [33]. DMPC in the DCM phase also had stabilization effects at the DCM/water interface. This may be due to an accumulation of DMPC at the water/DCM due to the presence of both hydrophilic and hydrophobic chains in DMPC, thus decreasing exenatide adsorption to the water/DCM interface [34]. 

Interestingly, poloxamer 188 improved stability at the DCM/water interface when dissolved in the water phase but had no effect when dissolved in DCM. The possible explanation can be found in the chemical structure of poloxamer 188. They are tri-block copolymers of the poly(ethylene oxide) (PEO) and poly(propylene oxide) (PPO). In the water phase, it is expected that the hydrophobic PPO and hydrophilic PEO will face the DCM and water phase, respectively, and be arranged with high affinity at the W/O interface. Therefore, exenatide in the water phase may be prevented sterically from contacting with DCM. In addition, there may be a stabilizing effect by micelle formation, but no further experiment for this micelle formation was performed in this study [44]. In contrast, both PPO and PEO parts could be retained in DCM when dissolved in the DCM phase because it is known that not only hydrophobic PPO but also hydrophilic PEO are soluble in DCM. Thus, exenatide could be exposed to DCM, thus destabilization explains their low efficacy in the inhibition of exenatide destabilization at the water/DCM interface [46]. 

**Figure 1:**
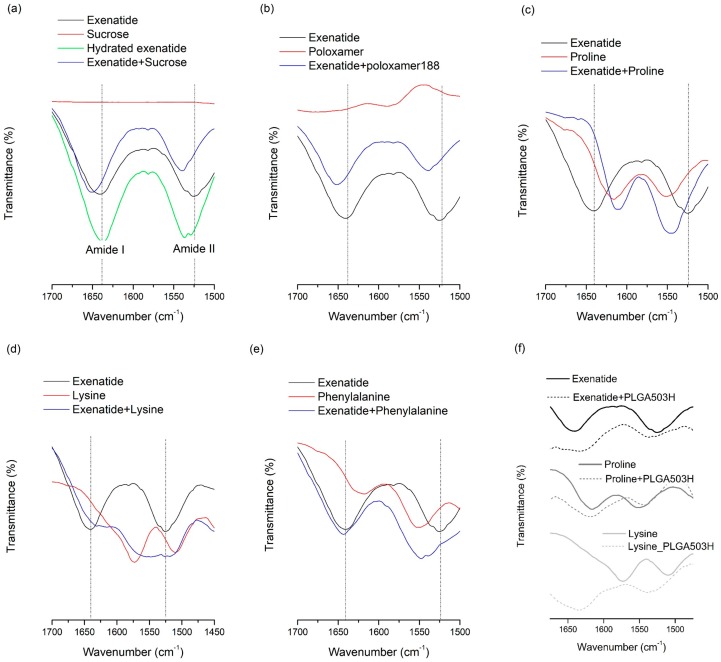
FT-IR spectra of exenatide with additives or PLGA: (**a**) exenatide and sucrose; (**b**) exenatide and poloxamer 188; (**c**) exenatide and proline; (**d**) exenatide and lysine; (**e**) exenatide and phenylalanine; and (**f**) PLGA with exenatide or proline or lysine.

FT-IR analysis was conducted for lyophilized samples containing both additives and exenatide to determine whether molecular interactions, such as a hydrogen bond, occurred [8,47,48,49]. The hydrogen bond-induced spectral changes can be seen in the region from 1700 to 1500 cm^−1^, which contains bands assigned to amide I and II of exenatide (Figure 1). All stabilizers added to the water phase showed remarkable changes, which indicate that there may be a specific interaction between exenatide and the stabilizer through the amide group. This result could mean that the molecular interaction of the stabilizer with the exenatide can help to improve stability at the W/O interface. Interestingly, there was no observed interaction between phenylalanine and exenatide in the FT-IR spectra (Figure 1e), which may mean the stabilization effect of phenylalanine at the W/O interface is caused solely by the affinity to the W/O interface, not by interaction with the exenatide. Therefore, it is suggested that the stabilization effect by the affinity to the W/O interface is the more dominant W/O interface stabilization mechanism than an interaction between the stabilizer and exenatide.

#### 3.1.3. Freeze-Thawing Stability

The recovery rate of 74.4% of exenatide after freeze-thawing indicates that freeze-thawing stress induces destabilization of exenatide in aqueous solution. This freeze-thawing stability is very important because destabilization of peptides occurs frequently due to freeze and freeze-thaw stresses during processing, storage, and transport of the drug product containing peptide. Freezing of a peptide solution may cause protein aggregation due the low temperature, solute concentration, formation of ice–water interfaces, and potential pH changes or phase separation [7]. 

**Figure 2:**
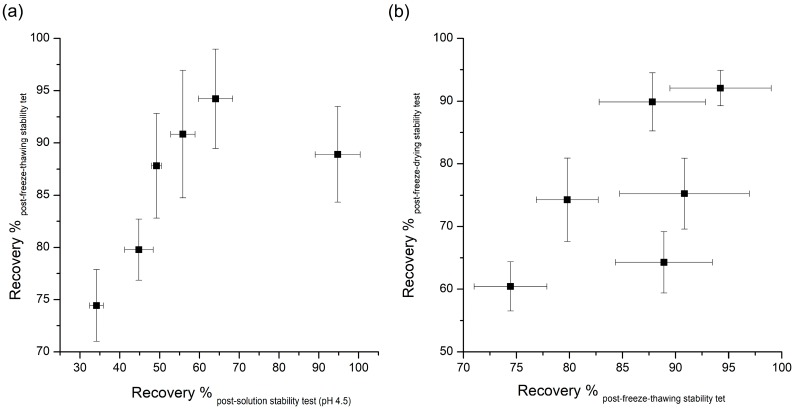
Correlation of the recovery rate (%) between: (**a**) pH 4.5 solution stability and freeze-thawing stability; and (**b**) freeze-thawing and freeze-drying stability.

Table 1 demonstrates that most additives tested can significantly prevent instability due to freeze-thawing, with sucrose causing the largest amount of stabilization. Many researches have been done on the effects of additives to improve the destabilization of peptides by freeze thawing. Many of the additives that improve peptides’ freeze-thawing stability can protect peptides from destabilizing by the preferential interaction mentioned in the aqueous stability section above [8]. As peptides are excluded from ice crystals during freezing, chemical and physical changes of peptides can occur in non-ice phases. In this pathway, cryoprotectants, such as the additives used in this study, can inhibit the destabilization of peptides through the preferential exclusion mechanism. The FT-IR results can explain the stabilization effect of stabilizers used in this study against freeze-thawing stress. Changes in the FT-IR spectra due to interactions between exenatide and stabilizers show the possibility of protecting the exenatide in place of water during freezing. As can be inferred by this theory, the significant changes in FT-IR spectra were observed in the exenatide coexisting with stabilizers, improving freeze-thawing stability, whereas the FT-IR spectrum was not observed for ineffective phenylalanine. These results further demonstrate that preferential interaction resulting from molecular interactions between exenatide and stabilizers are the major mechanisms governing freeze-thawing stability. In addition, several other mechanisms, such as increased solution viscosity, steric hindrance, or suppression of pH change during freezing, have been proposed for the explanation of the freeze-thawing stability enhancement effect [2]. Interestingly, the recovery rate obtained through pH 4.5 aqueous stability and freeze-thawing stability showed a pretty good correlation except for the results of poloxamer 188 (Figure 2a). This correlation shows that any factor altering peptide stability in aqueous solutions will tend to have the same qualitative effect during freeze thawing [8]. Although an enhancement effect for freeze-thawing-induced destabilization is due to the complex action of the various mechanisms mentioned above, it has been confirmed that the freeze-thawing stabilization effect by preferential interaction is most dominant, as reported earlier for other peptides and proteins.

#### 3.1.4. Freeze-Drying Stability

Exenatide recovery after lyophilization was 60.4%, which means the freeze-drying process can induce severe destabilization of exenatide. Lyophilization often induces stability issues because of the conformational instabilities in peptides that arise from both freezing and dehydration stresses. These two fundamentally different stresses should be accounted for when trying to improve freeze-drying stability. In contrast, recovery after lyophilization of sucrose and lysine were 92.1% and 89.9%, respectively, which indicates that both sucrose and lysine significantly enhance the freeze-drying stability of exenatide. This may be due to hydrogen bonds forming between the dried protein and the additive, which acts as a co-solute and water substitute, when the hydration shell of the peptide is removed [2,7,8]. To replace water molecules, hydrogen bonds with polar groups on the peptide surface are required for the molecular structure of the additive. In order to investigate the hydrogen bonding between hydrophilic additives and exenatide, FT-IR analysis was performed for lyophilized samples containing both additives and exenatide (Figure 1). The spectrum of the hydrated exenatide was different to that of the dried exenatide in bands assigned to amide I and II of exenatide over the range from 1700 to 1500 cm^−1^ (Figure 1a). This spectral change is due to conformational stabilization by hydration [8,48]. This is in good agreement with previous reports and shows the structural stabilization of exenatide by hydration. FT-IR analysis results showed an apparent change in the spectra due to a coexistence with various stabilizers. However, the shift trend of two peaks was completely different between sucrose and lysine. This difference indicates that each sucrose and lysine forms its own different type of hydrogen bond when it is present with exenatide in a solid state. Although the types of hydrogen bonds were different between sucrose and lysine, both cases resulted in an improved stability of the peptide during lyophilization. Unexpectedly, the addition of poloxamer 188 did not significantly improve the freeze-drying stability although both of them did enhance the aqueous and freeze-thawing stabilities with a significant change in FT-IR spectra due to an interaction with exenatide. The non-effect of poloxamer 188 for lyophilization stability is presumed to have more complicated reasons.

There was no correlation between the recovery rate of post-freeze-thawing and post-freeze-drying stability tests, which means the requirements for protection against dehydration during lyophilization are much more complicated and specific, and cannot be explained fully by only the preferential interaction mechanism (Figure 2b).

### 3.2. Effects of Additives on Adsorption of Exenatide to PLGA

#### 3.2.1. Effect of pH

**Figure 3:**
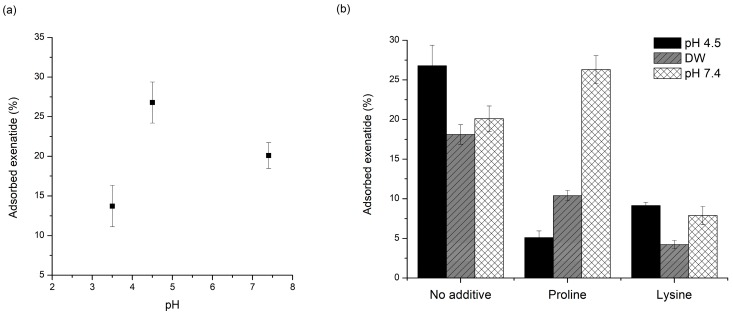
Effect of pH on exenatide adsorption onto a PLGA surface: (**a**) effect of PH without additives; (**b**) effect of PH with and without proline or lysine.

Figure 3a shows the pH-dependent adsorption of exenatide to PLGA. Adsorbed amounts of exenatide to the PLGA polymer were 13.7%, 26.8%, and 20.1% in pH 3.5, 4.5, and 7.4, respectively. The amount of adsorbed exenatide was the highest at pH 4.5, and this pH is very close to pI = 4.2 (isoelectric point) of exenatide [20,50]. Peptides and proteins are adsorbed more favorably to non-polar, high surface tension and charged than polar, low surface tension, and uncharged, respectively. Interactions with nonpolar and hydrophobic surfaces can lead to conformational reorientation through inter-peptide or peptide–surface interactions, thereby facilitating destabilization of peptides [51]. This kind of interaction with nonpolar and hydrophobic surfaces is directly related to nonspecific adsorption of peptide to the PLGA polymer. Nonspecific adsorption to the PLGA polymer appears to be greater at pH values closer to the pI of a peptide. At pH close to pI, the numbers of negative and positive charges are in balance, resulting in a net neutral-charged molecule, hence non-polar hydrophobicity is maximized. In addition, peptide molecules approach each other more closely and form more compact conformations, resulting in more effective adsorption. Adsorption rates are faster when the peptide and substrate bear opposite charges because electrostatic attraction can accelerate migration towards the surface. However, maximum total adsorption is generally observed at pI [52]. 

In contrast, the amount of adsorbed exenatide was the lowest at pH 3.5 below pI. At pH values below the pI, peptides are positively charged and exist in an unfolded form, resulting in repulsion between peptide molecules and requiring a larger space to overcome the repulsive forces generated. If the substrate has a positive charge, the adsorption would increase. However, pH 3.5 of the solution tested was lower than pKa (=3.85) of PLGA carboxylate, and the incubation time may not be enough to proceed much of the ionization of the carboxyl end group of PLGA [53]. For these reasons, the increase in adsorption due to charge interactions between exenatide and PLGA is not expected and rather decreases non-specific adsorption, which is supposed to have resulted in a reduction of total adsorption. 

The amount of adsorbed exenatide was decreased by an increase in pH to 7.4 over pI, but it was higher than that of pH 3.5. At pH values higher than pI, peptides are negatively charged, hence the repulsion between peptide molecules increases. At pH 7.4, even though the incubation was short, ionization of carboxylic acid groups could occur by hydration because used PLGA503H is uncapped PLGA. A negative or neutral net charge of the peptide could mask the fact that positively charged residues can still exist and may interact electrostatically with negatively charged carboxylic acid end-groups of PLGA [54]. Nevertheless, it is natural that non-specific interactions are reduced. This theory may explain why the adsorption at pH 7.4 increased than pH 3.5 and decreased than pH 4.5. These adsorption results indicate the critical role of ionized carboxylic acid end groups in PLGA during peptide sorption. 

The interaction of peptide drugs with PLGA can improve the encapsulation efficiency of those drugs, although such interactions can also cause problems during drug release from microspheres [55]. An initial burst release followed by an incomplete release of peptide from microspheres is frequently observed due to instability issues regarding acylation, with peptide adsorption to PLGA believed to be a common precursor to peptide acylation [1,6]. To obtain an ideal formulation possessing a lower burst release, optimal accumulative release, and complete release, various interactions between peptides and PLGA, especially during adsorption, must be considered [56]. The chemical interaction with acidic surfaces of degraded PLGA microspheres after the nonspecific adsorption of peptides during initial drug release severely limits the amount of peptide available for release, resulting in slow and incomplete release profiles [57]. In addition, instability due to acylation is particularly severe for positively charged peptides. The possibility of nonspecific peptide adsorption to and interactions with PLGA polymers should therefore be minimized in order to obtain a complete peptide release profile. The results of adsorption experiments in this study are not the stages in which acylation due to ionic interaction is obvious, but more closely related to the initial adsorption stage due to nonspecific interaction that occurs mainly during the initial hydration process. This result may not be consistent with the trend of change in stability by acylation. Therefore, further studies on the stability of acylation are needed. 

#### 3.2.2. Effects of Hydrophilic Additives

The prevention of adsorption of exenatide to PLGA by various additives was evaluated in aqueous solution at pH 4.5. Proline, lysine (Table 2), and poloxamer 188 significantly reduced exenatide adsorption from 26.8% to 5.1%, 9.1%, and 3.7%, respectively. Poloxamers 188 have been frequently used to minimize peptide adsorption onto hydrophobic surfaces due to their active surface properties and biocompatibility. The hydrophobic PPO is located at the center of the molecular structure, and they can attach to the PLGA surface by hydrophobic interaction. In addition, the longer PEO tails can act as steric barriers between the surfaces and the peptide molecules, hence the adsorption of the peptide could be inhibited [34]. 

Proline and lysine were effective in preventing exenatide from adsorbing to PLGA, whereas phenylalanine was ineffective. In the FT-IR spectra of exenatide mixtures with amino acids obtained by lyophilization, proline and lysine showed shifts in the amide peak due to interaction with exenatide, but phenylalanine showed no significant change (Figure 1). Through this, it can be estimated that amino acids, such as lysine and proline, may prevent direct adsorption of exenatide to PLGA by binding through molecular interaction with exenatide in the water phase. In addition, the inhibition of exenatide adsorption by the interaction of amino acids with PLGA were also considered. Both proline (pI = 6.3) and lysine (pI = 9.74) have a positive net charge at pH 4.5. Therefore, they could be rapidly adsorbed by specific interaction with the carboxyl group of PLGA than non-specific adsorption of exenatide. In order to evaluate molecular interactions with PLGA and amino acids, FT-IR analysis was performed on solid samples obtained after lyophilization by collecting PLGA layers after adsorption tests of amino acids in water without exenatide. Figure 1 shows the FT-IR spectra of unprocessed PLGA and PLGA adsorbed with amino acids. Interestingly, lysine showed a peak shift in the amide bands by interaction with PLGA, but proline did not show a significant peak shift. Therefore, it is estimated that the main mechanism of proline for the prevention of exenatide adsorption is molecular interaction with exenatide. It is supposed that lysine has two mechanisms of not only molecular interaction with exenatide but also a role as a barrier on the surface of PLGA due to a faster adsorption rate than exenatide. 

As mentioned above, this adsorption is a very important factor that greatly affects the stability of the peptide during the manufacturing process and drug release [2,58]. During the process, the peptide present in the internal water of the emulsion may be strongly adsorbed to PLGA fabricated at the W/O interface and may not be released properly. The peptides exiting the water phase through the water channels may be adsorbed onto the PLGA surface, resulting in an initial burst release. In addition, the adsorbed peptide onto the PLGA surface after release can be destabilized by acylation and acidic hydrolysis. Thus, the pH conditions in each process where adsorption can occur are different. Considering these issues, adsorption experiments were also performed at DW and pH 7.4. The pH 4.5 acetate buffer is the pH environment of the inner water phase during microsphere preparation. On the other hand, the adsorption test in water was performed taking into account the environment of the outer water phase during microsphere preparation [1]. The PBS pH 7.4 was selected considering the pH condition of in vitro release tests and actual physiology. Excluding poloxamer 188 of non-ionic surfactant, proline and lysine were used to evaluate the effect of pH on the change in adsorption prevention. Exenatide test solutions without additives were also evaluated for comparison. As shown in Figure 3b, adsorption of exenatide to PLGA in proline test solution increased in water and pH 7.4 compared to pH 4.5. Especially, at pH 7.4, the adsorption by proline was higher than that of the test solution containing only exenatide. Before the adsorption test, the fresh DW had a pH of almost 7, but after the adsorption evaluation, it dropped to near 6. In this condition, proline with pI of 6.3 will have a net charge near zero, hence the interaction between the charged or ionized groups of the proline and exenatide could be reduced and the adsorption protection effect is estimated to be reduced [59]. The reduction may be given even greater at pH 7.4, where the proline has negative charge. In contrast, lysine showed good adsorption protection in DW and pH 7.4. Since the lysine has a pI of 9.74, it has a positive charge at all pHs used in this experiment and a protective effect by interaction with exenatide having a negative net charge may be maintained. In addition, at pH 7.4, some carboxyl groups of PLGA can be ionized to a negative charge even for a short test time [60]. This may be another reason for maintaining the adsorption protection effect because lysine can be adsorbed relatively faster than exenatide through opposite charge interaction.

#### 3.2.3. Effect of Amphipathic Additives Blended in PLGA

All PLGA-blended amphipathic additives tested had no effect of inhibiting the adsorption of exenatide to PLGA (Table 2). There was no significant difference in the adsorbed exenatide amount between with and without poloxamer in PLGA film. It is presumed that the PEO tail is hidden inside the PLGA layer and does not function as a steric barrier. It was shown that the addition of DMPC in the PLGA layer increased the adsorption of exenatide by about two times. This may be due to the increased hydrophobicity of the PLGA surface [34].

### 3.3. Effects of Additives on Pharmaceutical Characteristics of Exenatide-Loaded PLGA Microsphere 

Based on the results of the above various stability evaluations of exenatide in aqueous solution, we selected the additives to be added to each phase of the double emulsion and observed how they affected the final microsphere. Poloxamer 188, sucrose, proline, and phenylalanine were added separately or in combination to the inner water phase of pH 4.5 in consideration of water-induced instability, lyophilization stability, desirable release behaviors, and W/O interface stability, respectively (Table 1). Lysine had an anti-adsorption effect in both water and pH 7.4. Lysine was added to the external aqueous phase (1% PVA in DW) instead of the internal buffer phase (pH 4.5) to evaluate whether lysine can prevent burst release by exenatide distributed on the external surface of the PLGA microsphere. In addition, the effects of lysine on the inhibition of destabilization and lag time by adsorption of exenatide to the PLGA surface were also considered.

#### 3.3.1. Particle Size and Morphology

**Figure 4:**
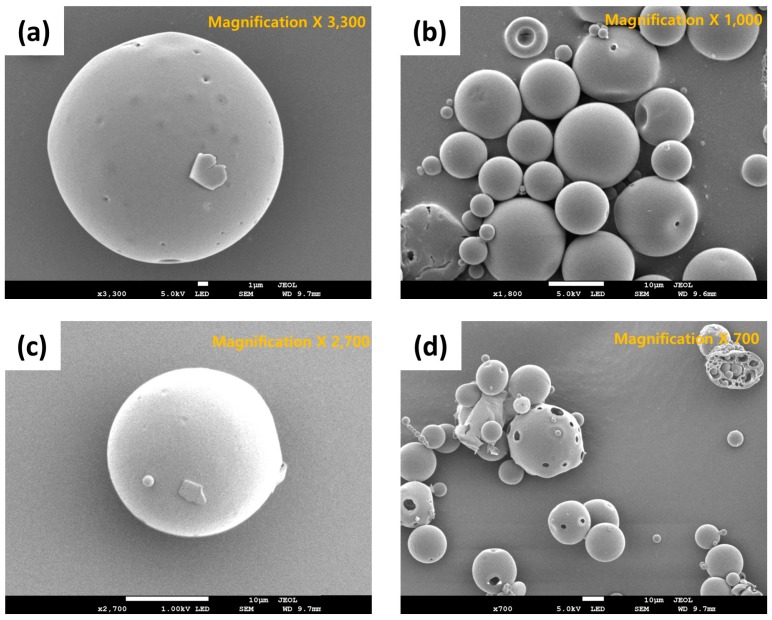
SEM images of exenatide-loaded PLGA microspheres (ELPM): (**a**) ELPM1; (**b**) ELPM5; (**c**) ELPM6; (**d**) ELPM7.

In this study, the volume mean diameter (VMD) and span value of exenatide-loaded PLGA microsphere (ELPM) were used to evaluate the particle size and its distribution. The VMD and span value of ELPM5 with lysine in the outer aqueous phase were smaller than that of ELPM1, which means the addition of lysine into the outer aqueous phase of the double emulsion resulted in a reduced particle size with a narrow distribution (Table 3). As discussed above, lysine does not have much surface activity, but this effect was very clear. It is assumed that there are more mechanisms, such as stabilization of the emulsion system by repulsion caused by charge at the O/W interface or an influence on the viscosity [3]. Further research is required to clarify this result. Except for the ELPM7 formulation, in which the poloxamer 188 was added to the inner aqueous phase, all formulations in which lysine was added to the outer aqueous phase were prepared as smooth surface microspheres of a uniform particle size distribution with a VMD of about 24 µm and a span value of less than 1.7. In contrast, the addition of poloxamer 188 to the outer aqueous phase creates surface porosity (Figure 4). It is presumed that the diffusion of poloxamer 188 from the DCM phase to the outer aqueous phase during the SE process can occur excessively and rapidly [61,62,63,64]. 

Particle size and surface morphology are important properties of microspheres and can affect degradation rates, drug loading, and the initial burst release of a drug. In the following section, we will address these correlations in detail for a discussion of the consequences of encapsulation efficiency (EE) and in vitro release.

#### 3.3.2. Encapsulation Efficiency (EE) and In Vitro Release of Exenatide-Loaded Microspheres

**Figure 5:**
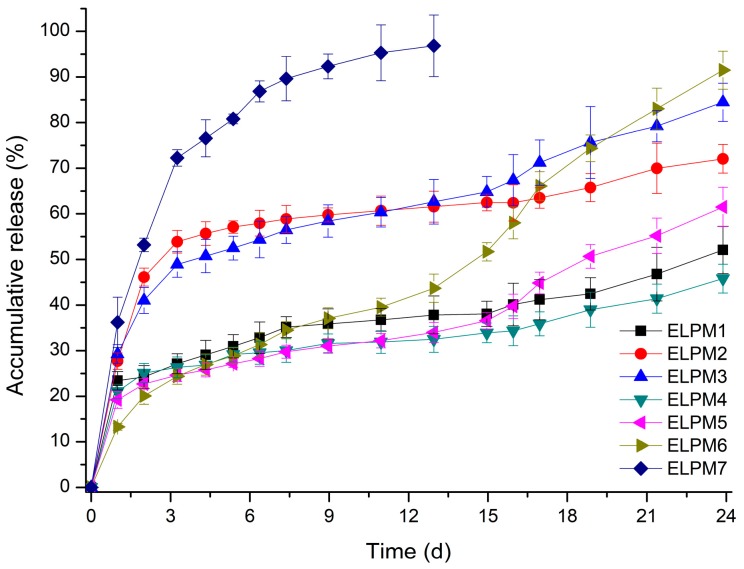
In vitro release profiles of exenatide-loaded PLGA microspheres (*n* = 3, pH 7.4 PBS, 37 °C).

Table 3 and Figure 5 show that the EE and release behavior of exenatide-loaded microspheres are influenced significantly by various additives. 

Comparing ELPM1 with ELPM2 and ELPM3, it was revealed that the addition of sucrose and proline to the inner aqueous phase resulted in a significant increase in EE. It is estimated that sucrose and proline can contribute to stabilization of exenatide in the internal water phase during the preparation process, as predicted by the stability evaluation results. In contrast, the initial burst release (IBR) of ELPM2 and ELPM3 increased slightly compared to ELPM1. This might be due to the higher osmotic pressure in the inner aqueous phase, as induced by the addition of sucorose or proline, enhancing the leakage of exenatide to the outer phase during the preparation process [65]. The addition of phenylalanine in the inner water phase for ELPM4 increased EE and decreased IBR compared to ELPM 2 and ELPM3. However, a severe lag time was observed after IBR for ELPM1, ELPM2 and ELPM4. For these three formulations, the total release was less than 17% from 7 to 24 days after the start of the release experiment. This is clearly because it does not prevent adsorption to PLGA and subsequent ionic interaction with the ionized carboxyl group of PLGA during the release period [20,56]. 

**Figure 6:**
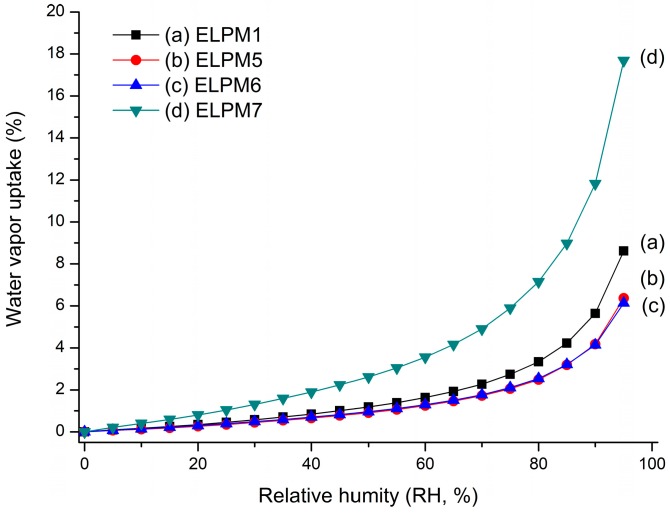
Water vapor sorption profile over increasing relative humidity (RH): (**a**) ELPM1; (**b**) ELPM5; (**c**) ELPM6; (**d**) ELPM7.

ELPM5 with lysine in the outer water phase showed a desirable effect of increasing EE and decreasing IBR despite decreasing the particle size. In particular, moisture sorption was reduced compared to ELPM1, as shown by the result of the water vapor sorption analysis (Figure 6). This result may indicate that EE increased due to the lower surface area and dense PLGA microsphere surface, and this positive effect for the surface was observed in the SEM, which shows that a small pore, which was rarely seen in the SEM image of ELPM1, was not observed in the SEM image of ELPM5 (Figure 4). 

Interestingly, ELPM3, ELPM5, and ELPM6 showed a decrease in lag time during drug release compare to ELPM 1, ELPM 2, and ELPM4. In order to clarify this difference, a representative comparison of ELPM2 and ELPM6 will be given. A typical biphasic release pattern was observed for ELPM 2, with an initial burst release followed by a more controlled secondary phase. However, the amount of exenatide release for ELPM2 was less than 10% from 3 to 17 days in the secondary phase. The final accumulated release percentage after 24 days was 72%, which indicates the incomplete release or destabilization of exenatide. Generally, ionic interaction may occur between peptide and carboxylic acid end-groups in PLGA, then non-specific adsorption on the degrading PLGA surface, covalent/non-covalent aggregation, and denaturation of peptide can be induced when the PLGA starts to degrade. These factors can cause the incomplete release of the drug from the PLGA microsphere. In addition, chemical degradation, such as acylation and deamination, could also occur during release. It is assumed that these factors are not controlled by the addition of sucrose alone, resulting in an undesirable release pattern. On the other hand, a typical triphasic release pattern was observed for ELPM6. After the initial burst release, the drug was released constantly for 24 days and the final accumulated release percentage after 24 days was 91%. The accelerated drug release in the third phase can be caused by a dramatic change of the conditions for the mass transport processes when the drug is still present as the original state without aggregation and/or destabilization by interaction with PLGA and the PLGA polymeric structure of the system becomes unstable and the macromolecular network breaks down. This in vitro release result is in good agreement with the results of the adsorption experiments and shows that proline and lysine can actually reduce the adsorption to PLGA and ionic interaction with PLGA during drug release at the inside and outside surfaces of the microsphere, respectively, hence improving the release profile through the stabilization of exnatide in the environment where release occurs. 

The decrease in EE of ELPM6 was expected compared to ELPM5 because sucrose and proline were added in the inner aqueous phase, and they can decrease EE due to the internal osmotic pressure. Interestingly, EE of ELPM6 did not decrease but was rather higher than that of ELPM5. The higher EE of ELPM6 than ELPM5 may be due to more destabilization during the microsphere preparation process because no additives with a protective effect were added to the inner aqueous phase of ELPM5. Moreover, IBR of ELPM6 was not only reduced compared to ELPM5, but exenatide was preferably released by more than 90% in 4 weeks due to the significantly reduced lag time. This result for ELPM6 shows that the positive role of lysine to the surface of PLGA microspheres, as shown in SEM images, can improve effectively the encapsulation of exenatide into the microsphere during the preparation process and the release behavior during the release period. As described in the adsorption test section, this positive effect of lysine may be due to the two mechanisms of not only molecular interaction with exenatide but also the role as a barrier on the surface of PLGA. Therefore, it was revealed obviously that the addition of proline to the internal water and the addition of lysine to the external water had a synergistic effect on the modification to a more desirable sustained release profile.

**Figure 7:**
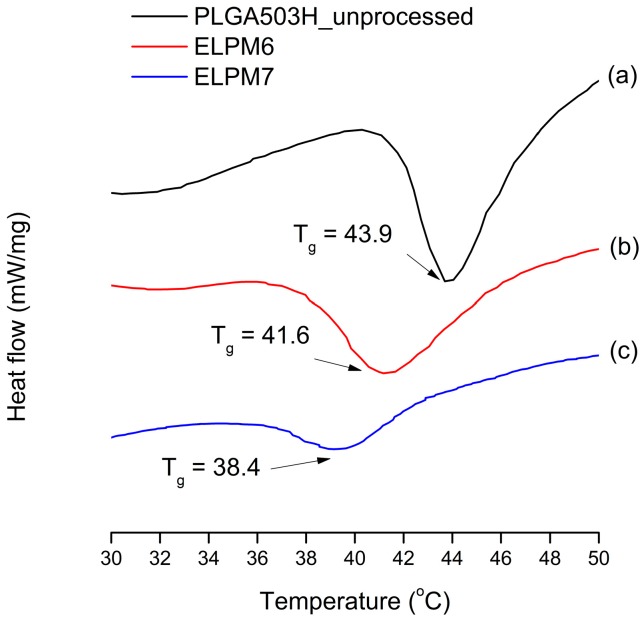
Change in glass transition temperature (*T_g_*) observed by DSC: (**a**) unprocessed PLGA; (**b**) ELPM6; (**c**) ELPM7.

For ELPM7, the addition of poloxamer 188 in the inner aqueous phase decreased the EE and increased the IBR. These negative effects of poloxamer 188 are potentially due to drug leakage and porous surfaces caused by surface activity of the surfactant and increases in surface-embedded peptides. Further support for this idea could come from porous surface measurements by SEM image analysis (Figure 4). In addition, the glass transition temperature (*T_g_*) decreased to 38.4 °C from 41.6 °C of ELPM6 (Figure 7), which indicates that PLGA with poloxamer 188 becomes more rubbery with larger mobility at physiological temperatures (37 °C) due to the plasticization effect of poloxamer 188 [66]. This plasticizer role of poloxamer 188 could explain why it was not possible to properly control the release of the drug during the manufacture of PLGA as well as during the release, thus resulting in low EE and high IBR for ELPM7. In contrast, no significant change in *T_g_* was observed when comparing ELPM6 with other formulations except ELPM7. However, the *T_g_* of ELPM6 was lowered by 41.6 °C compared to the *T_g_* = 43.9 °C of unprocessed raw PLGA. The heat and hydration during the SE process could reduce the *T_g_* of PLGA [55].

All results except poloxamer 188, were in good agreement with the results of the stability and adsorption experiments. Formulation optimization study through varying the number of additives should be carried out additionally.

#### 3.3.3. Secondary Structure Stability of Exenatide in PLGA Microsphere

**Figure 8:**
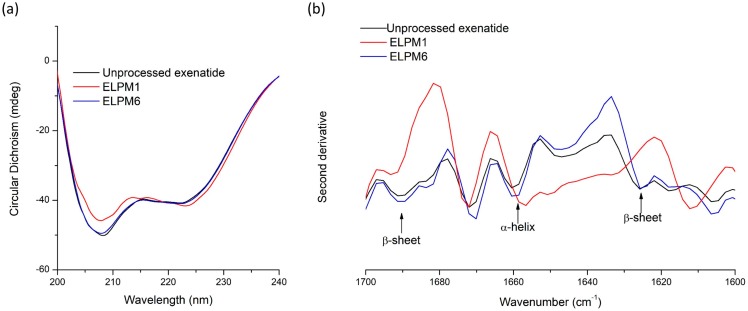
Secondary structure stability of unprocessed exenatide and extracted exenatide from ELPM1 and ELPM6: (**a**) circular dichroism spectra; (**b**) second derivative FT-IR spectra.

Unlike quantitative analysis of un-degraded exenatide using RP-HPLC, extracted exenatide from lyophilized microspheres was analyzed by circular dichroism (CD) spectroscopy to examine the changes in secondary structure in aqueous solution compared to unprocessed exenatide [12,18]. The CD spectra of exenatide had two specific minima at 209 and 224 nm wavelength, which indicated the presence of α-helix (Figure 8a) [25]. There was no difference between the extracted exenatide from ELPM6 and the unprocessed one, which means that the secondary structure of exenatide in ELPM6 microspheres can be maintained after the W/O/W–SE and lyophilization processes using a combination of various stabilizers [28,31]. On the other hand, for exenatide extracted from ELPM1 without protective additives in the formulation, a significant difference in the two minima at 209 and 224 nm was observed in the spectrum, implying a change in the secondary structure. This result shows that water soluble-destabilized exenatide in the secondary structure exists inside the ELPM1 microsphere [24].

To confirm the CD results and observe differences in the secondary structure in a solid state, second derivative FT-IR spectra were also obtained for unprocessed exenatide powder and exenatide extracted and lyophilized from ELPM1 and ELPM6 microspheres (Figure 8b). The peak at 1660 cm^−1^ in the amide I region is assigned to α-helix, and two peaks at 1692 cm^−1^ and 1629 cm^−1^ are identified as an intermolecular β-sheet [8,20,31]. These three peaks in second derivative FT-IR spectra can give useful information of the secondary structure of the peptide. There were significant differences in the peak assigned to α-helix between unprocessed exenatide and extracted exenatide from ELPM1, but not for extracted exenatide from ELPM6. Therefore, it was confirmed that ELPM6 prepared using a combination of stabilizers showed an enhanced stability of peptides in the microsphere.

## 4. Conclusions

In this study, we determined the effects of various additives, such as carbohydrates, amino acids, and surfactants, on the stability of exenatide in aqueous solutions, at water/DCM interfaces, and during freeze-thawing and freeze-drying procedures. Furthermore, we also examined the effects of additives on the adsorption of exenatide to the PLGA polymer. Stability data were well correlated to the EE of microspheres and the release behavior of exenatide from microspheres. Our selected formulation used a combination of additives based on the stability data and showed synergic improvement of EE and IBR. Consequently, this study demonstrates that the evaluation of various stability at the preformulation step is important in the development of PLGA microsphere injections containing peptides and proteins. This type of study can help to develop a formulation with a desirable quality of injectable PLGA microspheres for sustained release by predicting the stability and performance of the final formulation as well as stability issues in the manufacturing process.

## Figures and Tables

**Table 1:** Formulation of exenatide-loaded PLGA microsphere (ELPM).

Formulation	Inner Water Phase (W_1_, pH 4.5)			Oil Phase (DCM)		Outer Aqueous Phase (W_2_)		
	Exenatide (mg)	Additives	Volume (mL)	PLGA (mg)	Volume (mL)	PVA (%)	Lysine (M)	Volume (mL)
ELPM1	10		0.1	186	2.5	1		25
ELPM2	10	Sucrose (4 mg)	0.1	186	2.5	1		25
ELPM3	10	Proline (0.1 M)	0.1	186	2.5	1		25
ELPM4	10	Phenylalanine (0.1 M)	0.1	186	2.5	1		25
ELPM5	10		0.1	186	2.5	1	0.1	25
ELPM6	10	Sucrose (4 mg) Proline (0.1 M)	0.1	186	2.5	1	0.1	25
ELPM7	10	Sucrose (4 mg) Poloxamer188 (4 mg) Proline (0.1 M)	0.1	186	2.5	1	0.1	25

**Table 2:** Effect of various additives on exenatide stability.

Additive			Recovery (%) ± SD (n = 3)				Adsorption (%) to PLGA
Type	Name	Added Phase	Solution (pH 4.5)	W/O	Freeze-Thawing	Freeze-Drying	
Control	**-**	**-**	34.2 ± 1.7	59.4 ± 3.1	74.4 ± 3.4	60.4 ± 3.9	26.8 ± 2.6
Hydrophilic	Sucrose	Water	64.1 ± 4.3	62.1 ± 4.1	94.2 ± 4.8	92.1 ± 2.8	20.3 ± 1.7
	Proline	Water	55.8 ± 3.1	69.8 ± 2.6	90.8 ± 6.1	75.3 ± 4.6	5.1 ± 0.6
	Lysine	Water	49.2 ± 1.3	66.0 ± 3.0	87.8 ± 5.0	89.9 ± 4.6	9.1 ± 0.7
	Phenylalanine	Water	44.8 ± 3.6	99.2 ± 2.7	79.8 ± 2.9	74.3 ± 6.7	19.8 ± 1.9
Amphipathic	Poloxamer 188	Water	94.8 ± 5.6	70.7 ± 4.5	88.9 ± 4.6	64.3 ± 4.9	3.7 ± 0.4
		DCM		58.3 ± 3.7			
		PLGA					26.5 ± 2.0
	DMPC	DCM		70.9 ± 3.3			
		PLGA					43.8 ± 3.4

**Table 3:** Evaluated characteristics of prepared microspheres.

Formulation	VMD ^1^ (um)	Span ^2^	LC ^3^ (%)	EE ^4^ (%)	IBR ^5^ (%)
ELPM1	36.8 ± 3.2	2.0 ± 0.3	1.54 ± 0.04	30.3 ± 0.8	23.4 ± 2.1
ELPM2	38.8 ± 2.6	1.9 ± 0.1	2.26 ± 0.04	44.2 ± 0.7	27.7 ± 1.8
ELPM3	33.4 ± 2.1	2.0 ± 0.1	2.36 ± 0.08	46.3 ± 1.5	29.2 ± 2.1
ELPM4	34.9 ± 1.3	1.8 ± 0.1	2.68 ± 0.04	42.5 ± 0.8	21.0 ± 1.6
ELPM5	23.5 ± 1.6	1.6 ± 0.0	2.62 ± 0.06	51.3 ± 1.3	19.2 ± 1.9
ELPM6	23.6 ± 0.7	1.6 ± 0.0	3.14 ± 0.03	61.6 ± 0.6	13.3 ± 0.8
ELPM7	17.9 ± 0.4	1.6 ± 0.0	2.44 ± 0.15	47.8 ± 2.9	36.2 ± 5.5

^1^ Volume mean diameter, ^2^ Value calculated as the ratio of (D90%–D10%) to D50%, where DN% indicates the volume particle diameter at each cumulative volume percentage, ^3^ Loading capacity, ^4^ Encapsulation efficiency, ^5^ Initial burst release measured on the first day.

## Supplementary Materials

The following are available online at https://www.mdpi.com/1999-4923/11/12/627/s1, Figure S1: Chemical structures, Figure S2: Schematic representation of stability test methods.
