# Supplementary material for: Effect of Stabilizers on Encapsulation Efficiency and Release Behavior of Exenatide-Loaded PLGA Microsphere Prepared by the W/O/W Solvent Evaporation Method
Source: Pharmaceutics. 2019 Nov 24;11(12):627. doi: 10.3390/pharmaceutics11120627 (PMC6955873; doi:10.3390/pharmaceutics11120627)
Supplement: Supplementary file 1 [file pharmaceutics-11-00627-s001.pdf]

pharmaceutics

MDPI

Supplementary Materials: Effect of Stabilizers on Encapsulation Efficiency and Release Behavior of Exenatide loaded PLGA Microsphere Prepared by the W/O/W Solvent Evaporation Method

Heejun Park, Dong-Hyun Ha, Eun-Sol Ha, Jeong-Soo Kim, Min-Soo Kim and Sung-Joo Hwang

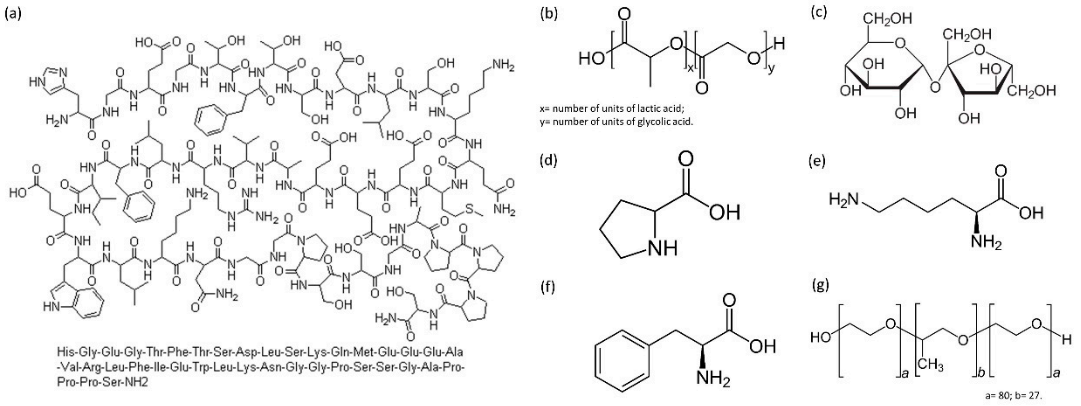

Figure S1. Chemical structures: (**a**) exenatide; (**b**) PLGA; (**c**) sucrose; (**d**) proline; (**e**) lysine; (**f**) phenylalanine; (**g**) poloxamer 188.

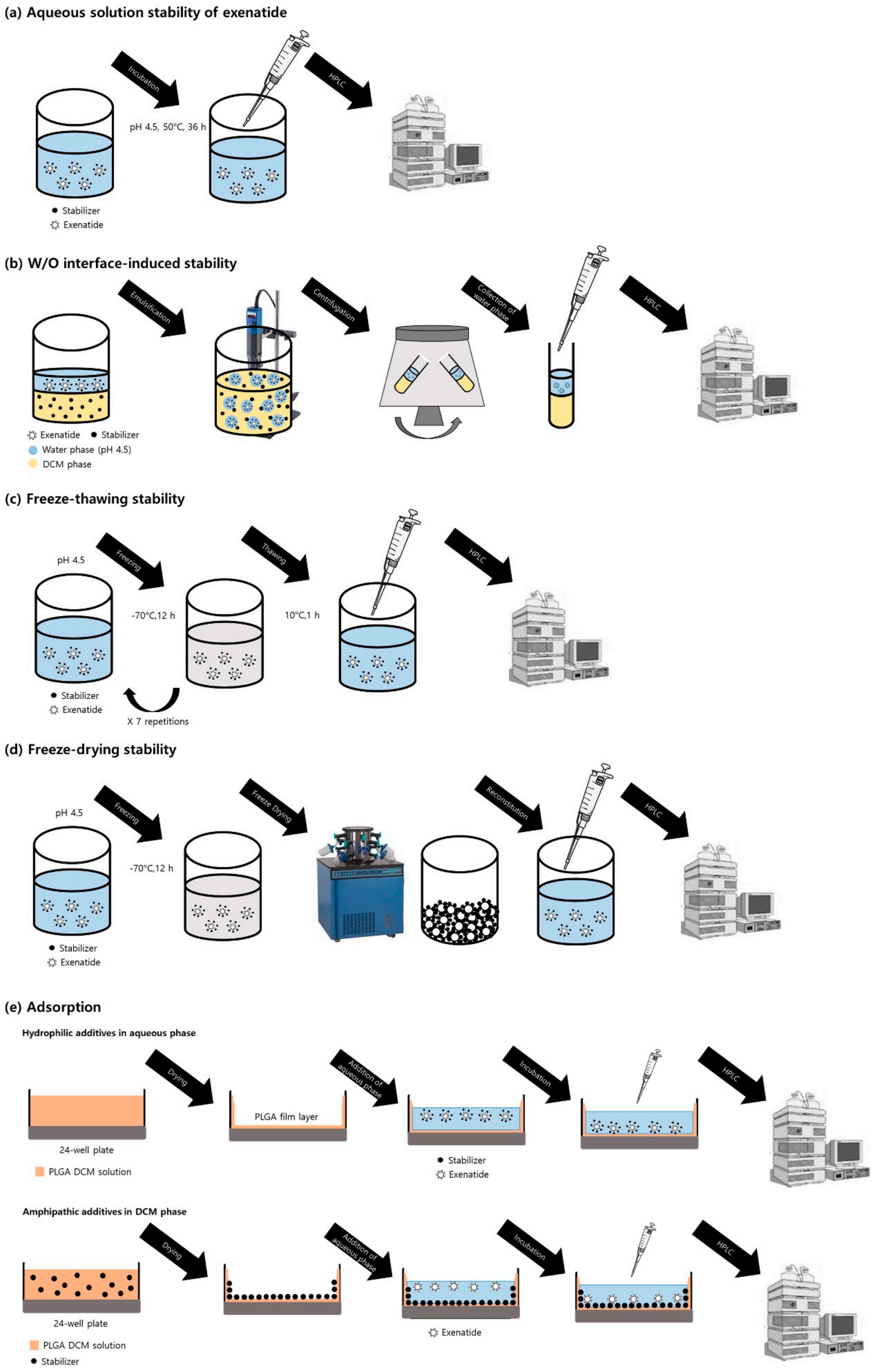

Figure S2. Schematic representation of stability test methods: (**a**) aqueous solution stability; (**b**) W/O interface-induced stability; (**c**) freeze-thawing stability; (**d**) freeze-drying stability; (**e**) adsorption.
